# The secret in their eyes: A review of the *recessus orbitalis*, a unique structure of flatfishes

**DOI:** 10.1111/joa.70000

**Published:** 2025-06-06

**Authors:** Paulo Presti, G. David Johnson, Aléssio Datovo

**Affiliations:** ^1^ Instituto de Biociências da Universidade de São Paulo São Paulo Brazil; ^2^ Museu de Zoologia da Universidade de São Paulo São Paulo Brazil; ^3^ National Museum of Natural History of the Smithsonian Institution Washington DC USA

**Keywords:** evolution, fishes, histology, Percomorpha

## Abstract

Flatfishes (Pleuronectiformes) are famous for having one of the most peculiar anatomical transformations in the animal kingdom, the ontogenetic migration of one eye from one side of the head to the other. But the eyes of flatfishes also carry a much lesser known but equally unique modification: an organ called the *recessus orbitalis*, which is responsible for projecting the eyeball above the level of the head, thus expanding their fields of vision. However, the morphology and distribution of the organ have never been thoroughly investigated. Previous studies reported only part of the *recessus orbitalis* and mistakenly suggested that it opened into the orbital cavity. We show that the organ forms a fully enclosed system consisting of two interconnected chambers: the facial chamber, which corresponds to the organ previously reported in the literature, and the scleral chamber, which encases the inner portions of the eyeball and is more challenging to detect through manual dissection. The organ is filled with interstitial fluid, and the walls of both chambers—especially the facial one—contain smooth, not skeletal and muscle fibers. These findings combined with field observations allowed us to propose a new functional model for the *recessus orbitalis*. The organ seems to operate as a dual‐pump system, dynamically shifting interstitial fluid between the facial and scleral chambers. Inflation of the facial chamber results in eye retraction, whereas inflation of the scleral chamber causes eye protrusion. The presence of smooth muscle fibers, which can sustain contractions with minimal energy expenditure, supports this inferred mechanism, allowing the eye to remain fully protruded or retracted for extended periods. The *recessus orbitalis* has never been confirmed in several flatfish families, and the organ was recently considered absent in Psettodidae, the sister group to all other flatfishes. However, we positively identified this organ in all 74 species examined representing all 16 currently recognized flatfish families, including Psettodidae. This indicates that the presence of the *recessus orbitalis* is an evolutionary novelty (synapomorphy) for the entire Pleuronectiformes.

## Graphical abstract

The *recessus orbitalis* is an organ responsible for projecting the eyeball above the level of the head and is uniquely present in flatfishes. The organ consists of two interconnected chambers filled with interstitial fluid and is internally coated with smooth muscle fibers that act as a double‐pump system.

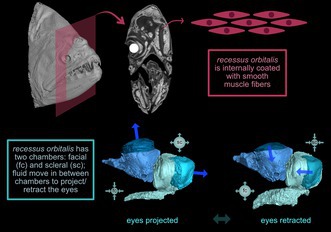

## 1 INTRODUCTION

Pleuronectiformes is an order with 16 families and 820 species (Fricke et al., 2025) that comprises all flatfishes, notably known to undergo one of the most peculiar morphological transformations during development: the migration of one eye from one side of the head to the other (Munroe, 2015; Schreiber, 2013). Due to this eye migration, adult pleuronectiforms have an asymmetrical neurocranium, reflecting on several other anatomical structures of their bodies (Friedman, 2008; Geffen et al., 2007; Gibson et al., 2015; Kyle, 1921; Nelson, 2006; Presti et al., 2024; Schreiber, 2013).

The intrarelationships of Pleuronectiformes were tested based on morphological (Chapleau, 1993; Hoshino, 2001) and molecular data (Azevedo et al., 2008; Berendzen & Dimmick, 2002; Betancur‐R, Broughton, et al., 2013; Betancur‐R, Li, et al., 2013; Campbell et al., 2013, 2014, 2019; Chen et al., 2003; Harrington et al., 2016; Hughes et al., 2018; Li et al., 2009; Near et al., 2013; Near & Thacker, 2024; Pardo et al., 2005), but only the work of Chapleau (1993) proposed morphological synapomorphies for the entire order. According to the author, there are three synapomorphies uniting all flatfishes into a monophyletic group: adults with an asymmetrical neurocranium, dorsal fin anteriorly advancing onto the neurocranium and the presence of the *recessus orbitalis*.

The *recessus orbitalis* is a fluid‐filled sac‐like muscular structure that protrudes the eyes by contracting its walls (Holt, 1894). Despite its supposedly synapomorphic status for the order, Chapleau (1993) stated that the occurrence of such a structure was only inferred and would still need to be confirmed in several subgroups of Pleuronectiformes. In addition, the *recessus orbitalis* was never properly illustrated in detail, appearing only in the form of a schematic diagram in Cole and Johnstone (1902) and in fresh specimen photographs in the recent work of Campbell et al. (2020). According to Campbell et al. (2020) the presence of the *recessus orbitalis* is a synapomorphic trait of Pleuronectoidei only, due to their conclusion that it is absent in *Psettodes*. Nevertheless, none of these studies proposed any kind of information on the type of muscular cell present in the organ, and its morphology remains poorly known for more than a century since its original description.

Here we present the first thorough morphological survey on the *recessus orbitalis*, discussing its anatomy and functional implications as well as documenting the distribution of this unique structure across all 16 families of Pleuronectiformes.

## 2 MATERIALS AND METHODS

This study was conducted under the approval of the Animal Care and Use Committee (ACUC) of the Instituto de Biociências, Universidade de São Paulo to A. Datovo (Projects #226/2015 and #446/2025; CIAEP #01.0165.2014). Anatomical nomenclature follows Presti et al. (2020) for osteology and Winterbottom (1974) for myology.

### 2.1 Regular dissections

Specimens were double stained following Datovo and Bockmann (2010) allowing the integrated view of bones, muscles, and nerves. Digital bidimensional color illustrations were produced with an XP‐Pen Artist 2. Material examined: Achiridae: *Achirus declivis* (MZUSP 116278), *Apionichthys dumerili* (MZUSP 51838), *Catathyridium jenynsii* (MZUSP 21147), *Gymnachirus nudus* (MZUSP 72718), *Hypoclinemus mentalis* (MZUSP 101594), *Trinectes paulistanus* (MZUSP 4353); Achiropsettidae: *Mancopsetta maculata* (USNM 362528); Bothidae: *Arnoglossus laterna* (USNM 282271), *Asterorhombus fijiensis* (USNM 362478), *Bothus lunatus* (MZUSP 71750), *Chascanopsetta lugubris* (USNM 282750), *Engyprosopon mogkii* (USNM 137961), *Monolene antillarum* (MZUSP 72275), *Parabothus chlorospilus* (USNM 375587), *Taeniopsetta radula* (USNM 375595), *Trichopsetta ventralis* (USNM 156078); Citharidae: *Brachypleura novaezeelandiae* (USNM 236122), *Citharoides macrolepidotus* (USNM 282958), *Citharus linguatula* (USNM 236123); Cyclopsettidae: *Citharichthys spilopterus* (MZUSP 71806), *Cyclopsetta chiittendeni* (MZUSP 72121), *Etropus crossotus* (MZUSP 118902), *Syacium micrurum* (MZUSP 51573), *Xystreuris rasilis* (MZUSP 72588); Cynoglossidae: *Cynoglossus lingua* (USNM 345455), *Paraplagusia bilineata* (USNM 273773), *Symphurus jenynsi* (MZUSP 12878); Oncopteridae: *Oncopterus darwinii* (MZUSP 10030); Paralichthodidae: *Paralichthodes algoensis* (SAIAB 118830); Paralichthyidae: *Ancylopsetta quadrocellata* (USNM 118487), *Paralichthys isosceles* (MZUSP 91684); *Pseudorhombus arsius* (USNM 138000), *Tephrinectes sinensis* (USNM 86372); Pleuronectidae: *Atheresthes stomias* (USNM 266605), *Eopsetta jordani* (USNM 27118), *Glyptocephalus cynoglossus* (USNM 261527), *Hippoglossus hippoglossus* (USNM 163652), *Limanda ferruginea* (USNM 290994), *Lyosetta exilis* (USNM 60159), *Platichthys stellatus* (USNM 126860), *Pleuronectes platessa* (USNM 197577), *Pleuronichthys coenosus* (USNM 83871); Psettodidae: *Psettodes erumei* (MZUSP 63360), *Psettodes belcheri* (USNM 286356); Poecilopsettidae: *Nematops macrochirus* (AM I.22821–044), *Poecilopsetta albomarginata* (USNM 184975); Rhombosoleidae: *Ammotretis rostratus* (USNM 282708), *Pelotretis flavilatus* (USNM 318393), *Peltorhamphus novaezeelandiae* (USNM 410298), *Rhombosolea tapirina* (USNM 214726); Samaridae: *Plagiopsetta glossa* (NSMT 57485), *Samaris cristatus* (USNM 314335), *Samariscus triocellatus* (USNM 354432); Scophthalmidae: *Lepidorhombus whiffiagonis* (NRM 25254), *Scophthalmus rhombus* (USNM 28446), *Zeugopterus punctatus* (NRM 20692); and Soleidae: *Achiroides melanorhynchus* (USNM 230355), *Aesopia cornuta* (USNM 397274), *Aseraggodes dubius* (USNM 137672), *Brachirus orientallis* (MZUSP 63355), *Buglossidium luteum* (USNM 48289), *Liachirus melanospilos* (USNM 76657), *Microchirus variegatus* (USNM 286962), *Pardachirus marmoratus* (USNM 306420), *Pegusa lascaris* (USNM 286979), *Solea vulgaris* (USNM 39419), *Zebrias zebrinus* (NSMT 78329).

### 2.2 Microcomputed tomography

One specimen of *Psettodes erumei* (MZUSP 63360) was stained and scanned to analyze its cranial anatomy. The specimen was stained by full immersion in a 2% iodine solution with 70% ethanol for 7 days covered with foil to prevent photoreaction. The specimen was later rinsed in 70% ethanol and immersed in this solution for 1 day. Images were obtained with a microtomography Phoenix v|tome|x m microfocus of the General Electric Company. The image reconstructions were done by Phoenix datos| × 2 reconstruction; GE Sensing and Inspection Technologies GmbH and edited via VG Studio Max version 3.4 64bits, Volume Graphics GmbH.

### 2.3 Histology

Tissues from the head of 13 species from 10 families were decalcified in 20% formic acid (Humason, 1972), washed, and dehydrated for paraffin embedding. Sections were cut at 6 μm, and all slides were stained with Masson trichrome at the Cornell Veterinary College Diagnostic Laboratory. Images were obtained using a Nikon CiL Compound microscope with a 16.25‐megapixel Nikon DS‐RI2 Color Camera and NIS Elements software. Material examined: Achiridae: *Achirus lineatus* (USNM 156403); Bothidae: *Bothus ocellatus* (USNM 236249); Citharidae: *Citharus linguatula* (USNM 236123); Cyclopsettidae: *Citharichthys atlanticus* (USNM 155805); Cynoglossidae: *Symphurus elongatus* (USNM 394716), *Symphurus melanurus* (USNM 394644), *Cynoglossus carpenteri* (USNM 203998), *Cynoglossus puncticeps* (USNM 137686); Pleuronectidae: *Lyopsetta exilis* (USNM 60159); Poecilopsettidae: *Poecilopsetta albomarginata* (USNM 184975); Psettodidae: *Psettodes erumei* (USNM 305276); Scophthalmidae: *Scophthalmus aquosus* (USNM 118218); Soleidae: *Solea vulgaris* (USNM 236109).

### 2.4 Field observation

One living individual of *Achirus lineatus* was used for observation. We stimulated one eye of *A. lineatus* in order to observe the natural response to an income threat and the subsequent retraction of the eye.

## 3 RESULTS

### 3.1 Morphology

**FIGURE 1:**
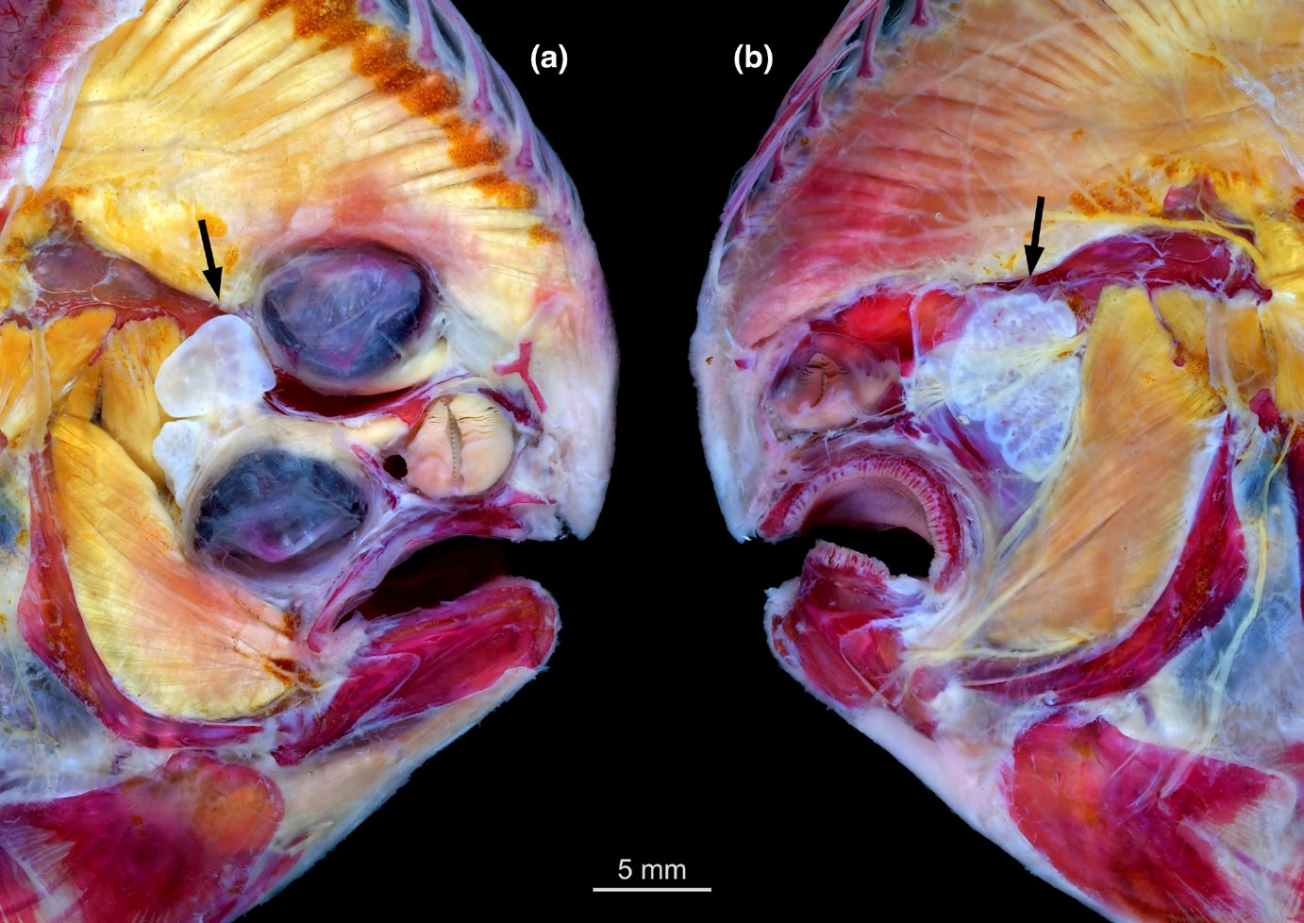
Head of *Pegusa lascaris* with superficial head musculature exposed. (a) ocular side and (b) blind side. Arrow indicating the facial chamber of the *recessus orbitalis* of both sides.

**FIGURE 2:**
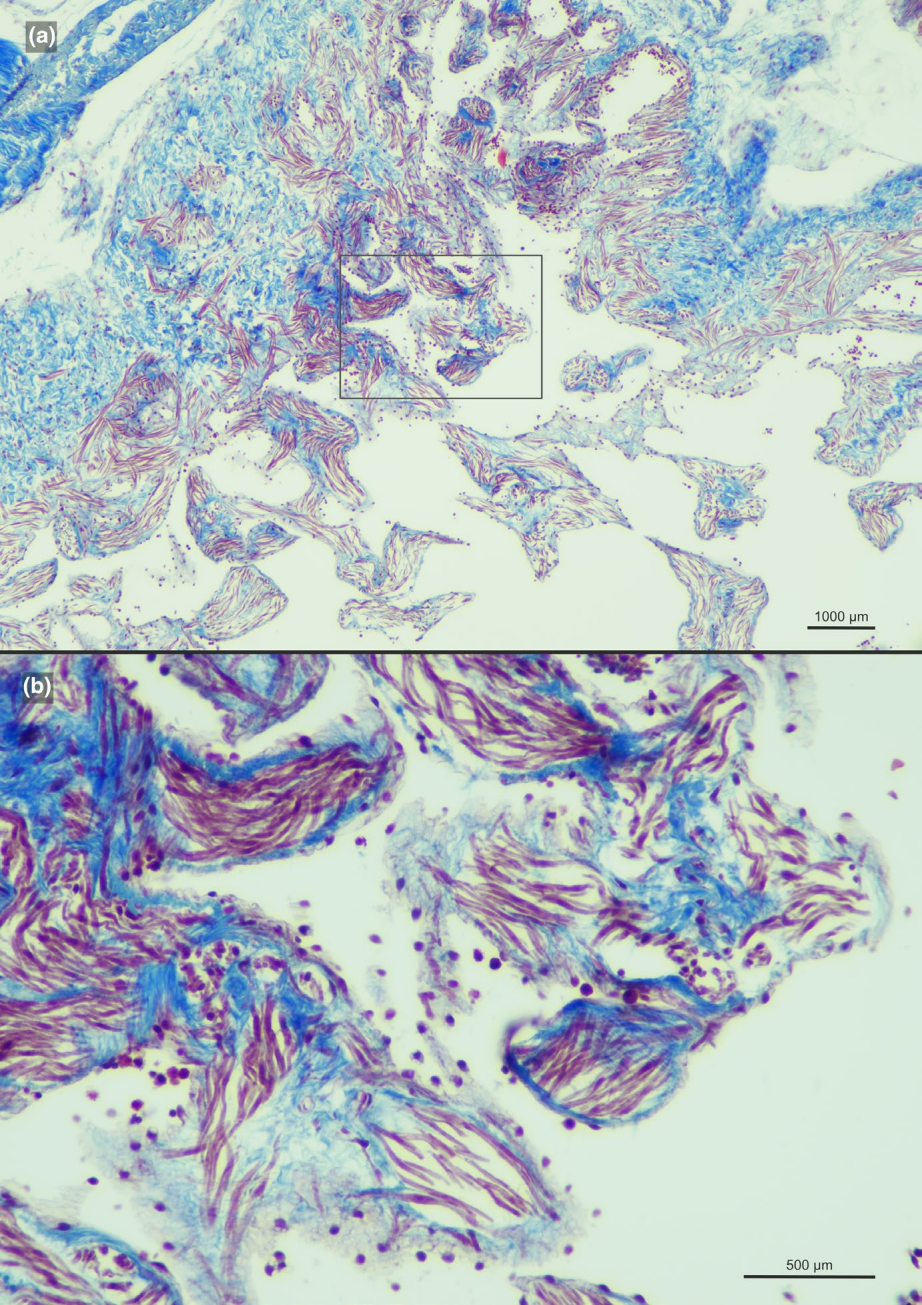
Internal anatomy of the facial chamber of the *recessus orbitalis* of the blind side of *Bothus ocellatus* showing the smooth muscle fibers in dark red. (a) longitudinal section, (b) zoom in of (a).

**FIGURE 3:**
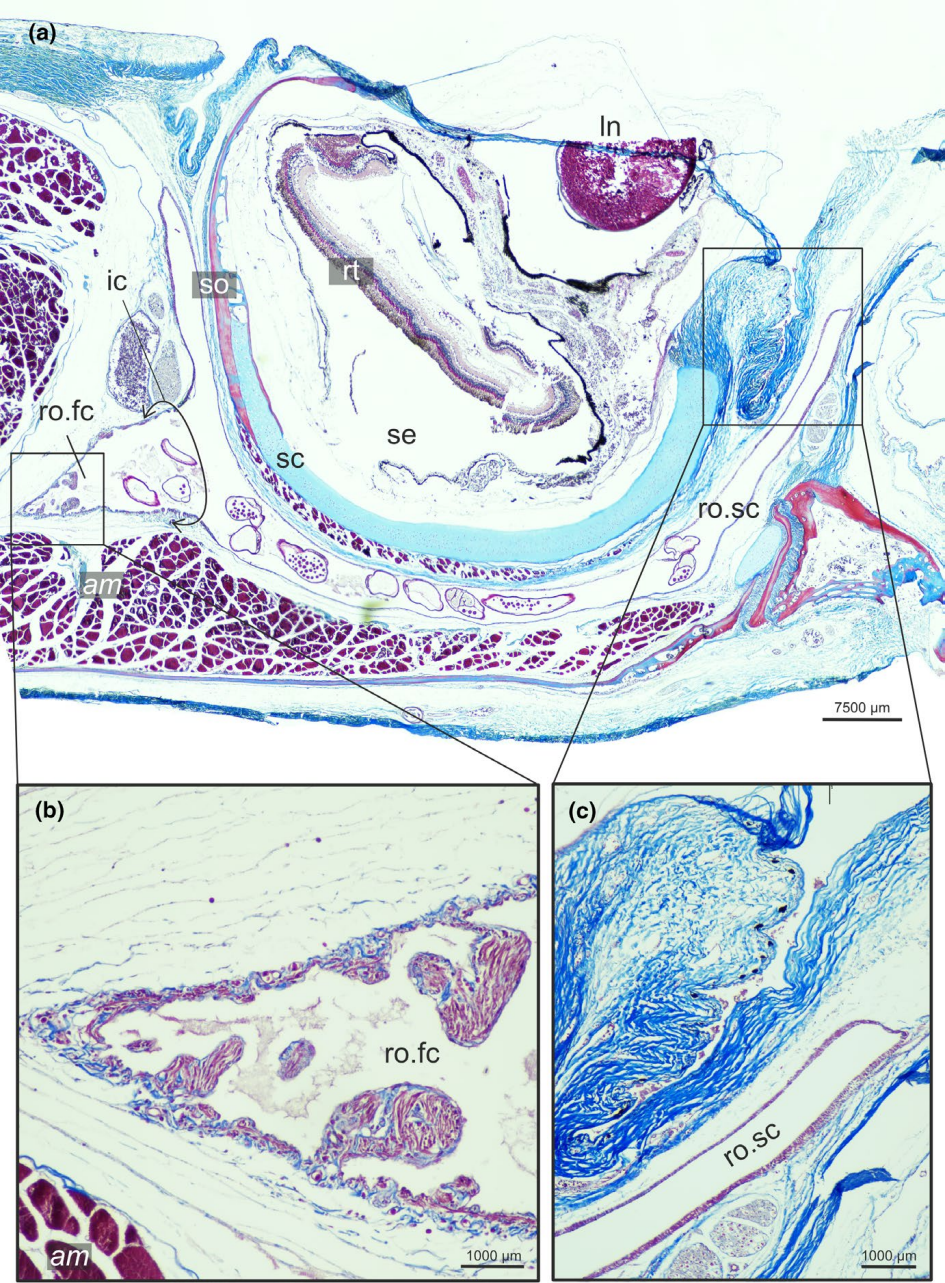
Sagittal section of the head of *Poecilopsetta albomarginata*. (a) Broad view of the section showing the sessile eye and its surrounding tissues. (b) Detail of the facial chamber of the *recessus orbitalis* and its internal smooth muscle fibers. (c) Detail of the margin of the eye showing the elastic nature of the rippled texture when the eye is retracted and the scleral chamber of the *recessus orbitalis*. am, facial segment of the *adductor mandibulae*; ln, lens; ro.fc, *recessus orbitalis*, facial chamber; ro.sc, *recessus orbitalis*, scleral chamber; rt., retina; sc, Sclera; se, Sessile eye; so, Sclerotic ossicle.

**FIGURE 4:**
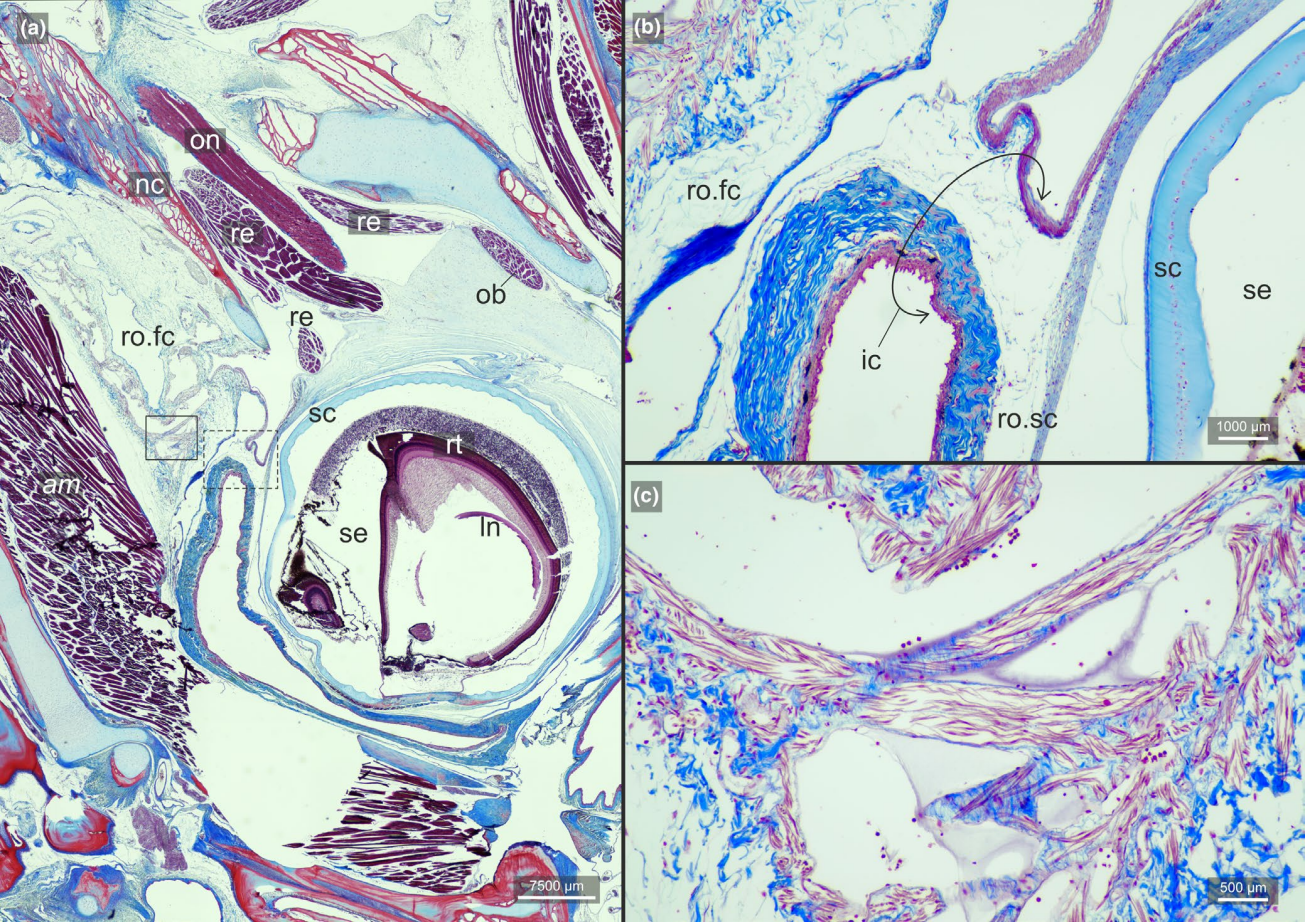
Longitudinal section of the ocular side of the head of *Bothus ocellatus*. (a) Broad view of the section showing the sessile eye and its surrounding tissues. Dashed‐line rectangle indicates the region of which (b) image zooms in. Continuous rectangle indicates the region of which (c) image zooms in. (b) Region in which facial chamber of the *recessus orbitalis* communicates with the scleral chamber of the *recessus orbitalis*. (c) Facial chamber of the *recessus orbitalis* and its internal smooth muscle fibers in red. am, facial segment of the *adductor mandibulae*; ic, interchamber constriction; ln, lens; nc, neurocranium; ob, *obliqui* muscles; on, optic nerve; re, *recti* muscles; ro.fc, *recessus orbitalis*, facial chamber; ro.sc, *recessus orbitalis*, scleral chamber; rt., retina; sc, sclera; se, sessile eye.

**FIGURE 5:**
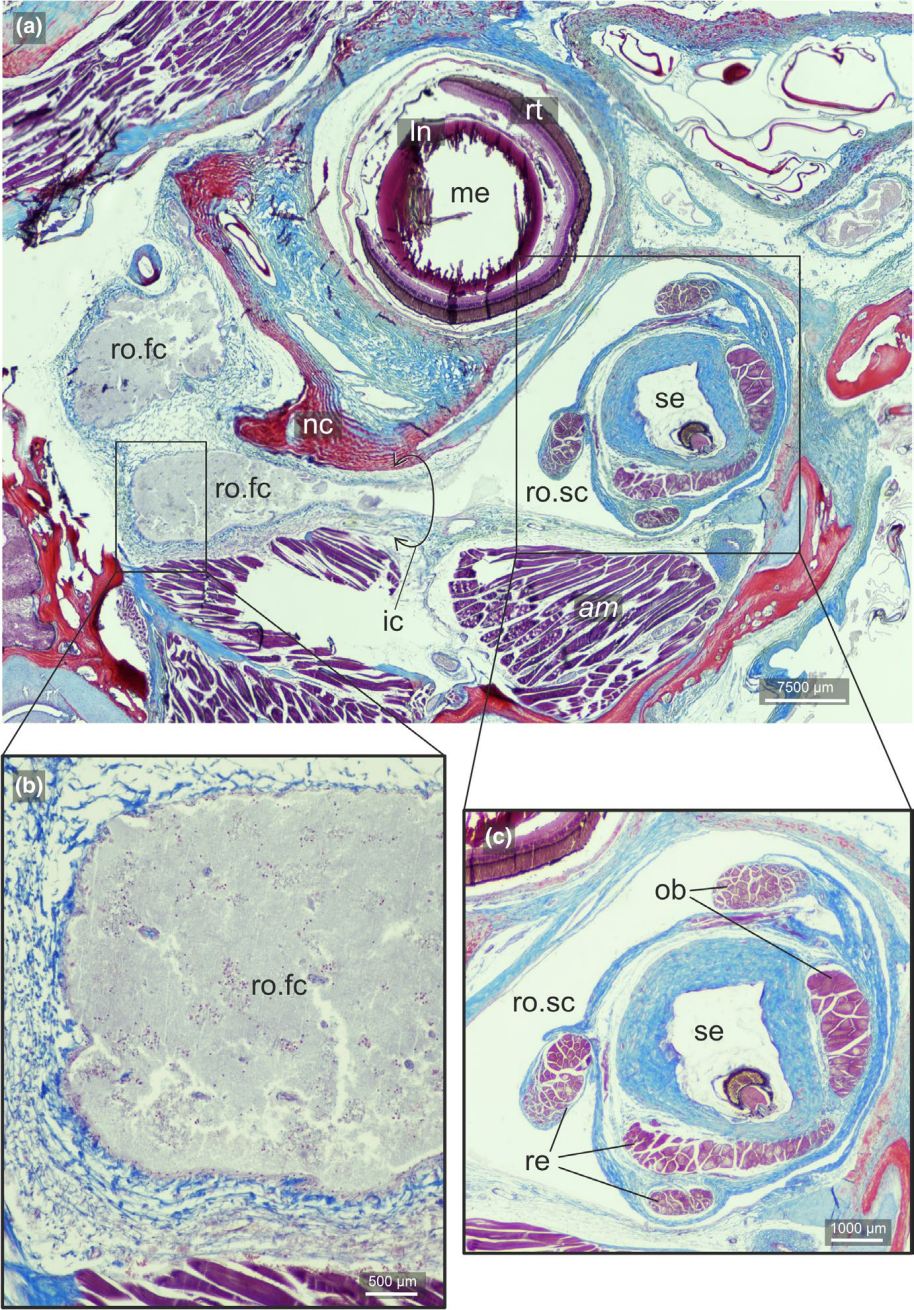
Longitudinal section of the ocular side of the head of *Symphurus melanurus*. (a) Broad view of the section showing the sessile eye and its surrounding tissues. (b) Detail of the facial chamber of the *recessus orbitalis*. (c) Detail of the sessile eye surrounded by the scleral chamber of the *recessus orbitalis*. am, Facial segment of the *adductor mandibulae*; ic, interchamber constriction; ln, lens; me, migrated eye; nc, neurocranium; ob, *obliqui* muscles; rc, Retraction chamber; re: *Recti* muscles; ro.fc: *Recessus orbitalis*, facial chamber; ro.sc: *Recessus orbitalis*, scleral chamber; rt.: Retina; sc: Sclera; se: Sessile eye.

Our survey included 74 species from all 16 flatfish families, and the *recessus orbitalis* was invariably found in both ocular and blind sides (Figure 1) of every taxon of Pleuronectiformes examined, including *Psettodes*. We discovered that the *recessus orbitalis* is covered in smooth muscle fibers (Figure 2) and is actually composed of two main thin‐walled chambers interconnected by an interchamber constriction (Figures 3, 4, 5). The scleral chamber surrounds and is associated with the external surface of the sclera. Smooth muscle fibers are present usually as a thin layer in the walls of the scleral chamber (Figure 3c). This chamber is superficially hidden by the eyeball and has been overlooked in all previous studies. Such studies have only reported the second, larger and pouch‐like chamber, referred to here as the facial chamber of the *recessus orbitalis*. This chamber typically has several invaginations that form small alveoli profusely filled with smooth muscle fibers (Figure 2). These alveoli communicate into a single atrium that converges to the interchamber constriction that communicates with the scleral chamber (Figures 3, 4, 5). Some cross sections show the facial chamber filled with a coagulated fluid (Figure 5). Both the ocular and blind *recessus orbitales* are innervated by branches of the *ramus maxillaris trigeminus*.

**FIGURE 6:**
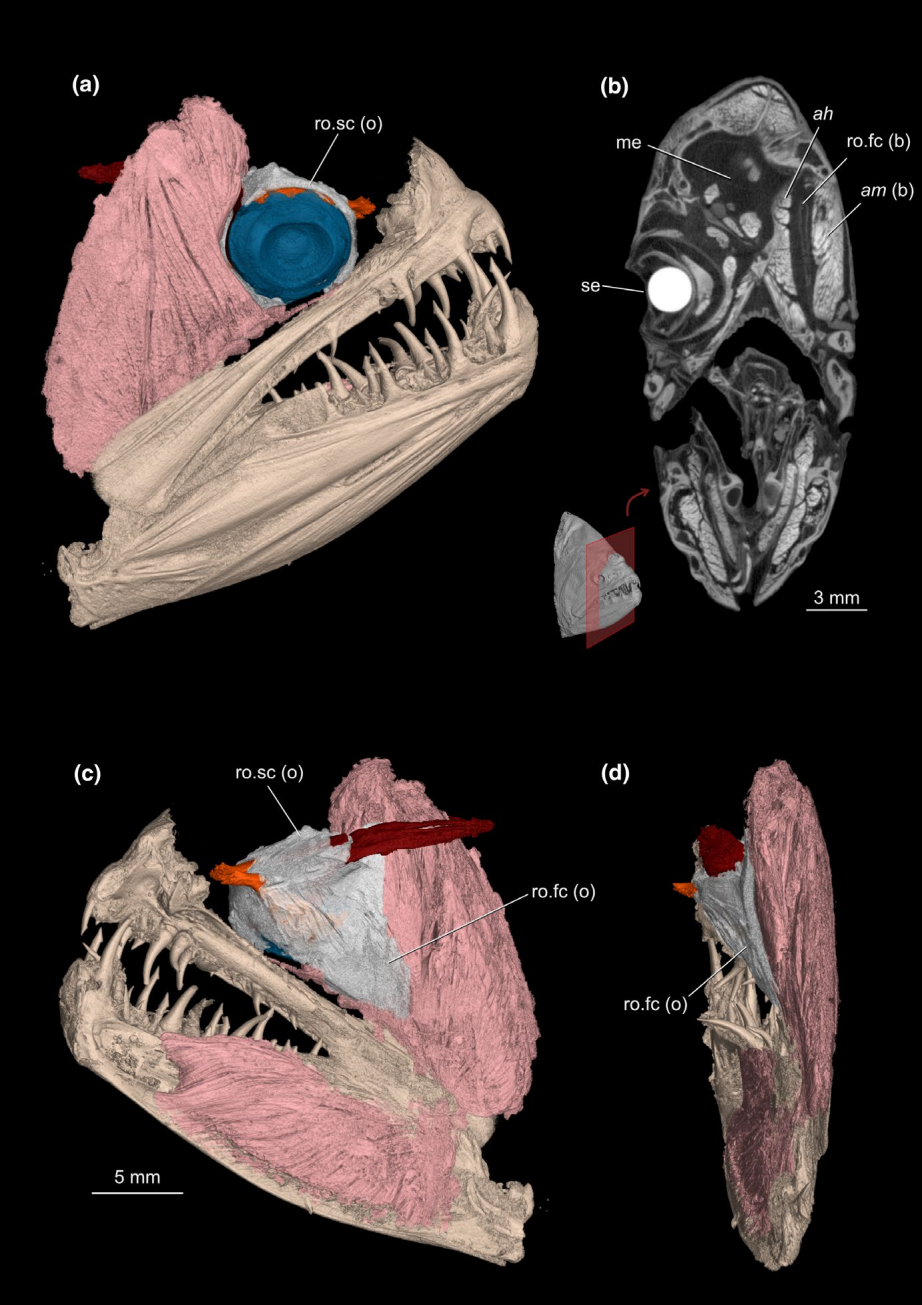
X‐ray microcomputed tomographies of the mandibular jaw of the ocular side of *Psettodes erumei* showing the facial segment of the *adductor mandibulae* and the sessile eye with its associated muscles and *recessus orbitalis*. (a) Lateral view, (b) transversal section of the head showing the position of the blind side *recessus orbitalis*, sandwiched in between the facial segment of the *adductor mandibulae* and *adductor hyomandibulae*, (c) medial view, (d) Posterior view. ah, *adductor hyomandibulae*; am, facial segment of the *adductor mandibulae*; (b) blind side; me, Migrated eye; (o) ocular side; ro.fc, *recessus orbitalis*, facial chamber; ro.sc, *recessus orbitalis*, scleral chamber; se, sessile eye.

While the scleral chamber of both sides has a nearly constant position and morphology across all flatfishes, the facial chamber shows some important variations. The facial chamber of the ocular side is located posterodorsally to the sessile eye, immediately ventral to the frontal bone (Figures 1a, 4 and 5). In some species, this chamber is so developed that it marks the posterior margin of the sessile eye, also occupying the medial space in between the facial segment of the *adductor mandibulae* and *adductor hyomandibulae* muscles (Figure 6).

**FIGURE 7:**
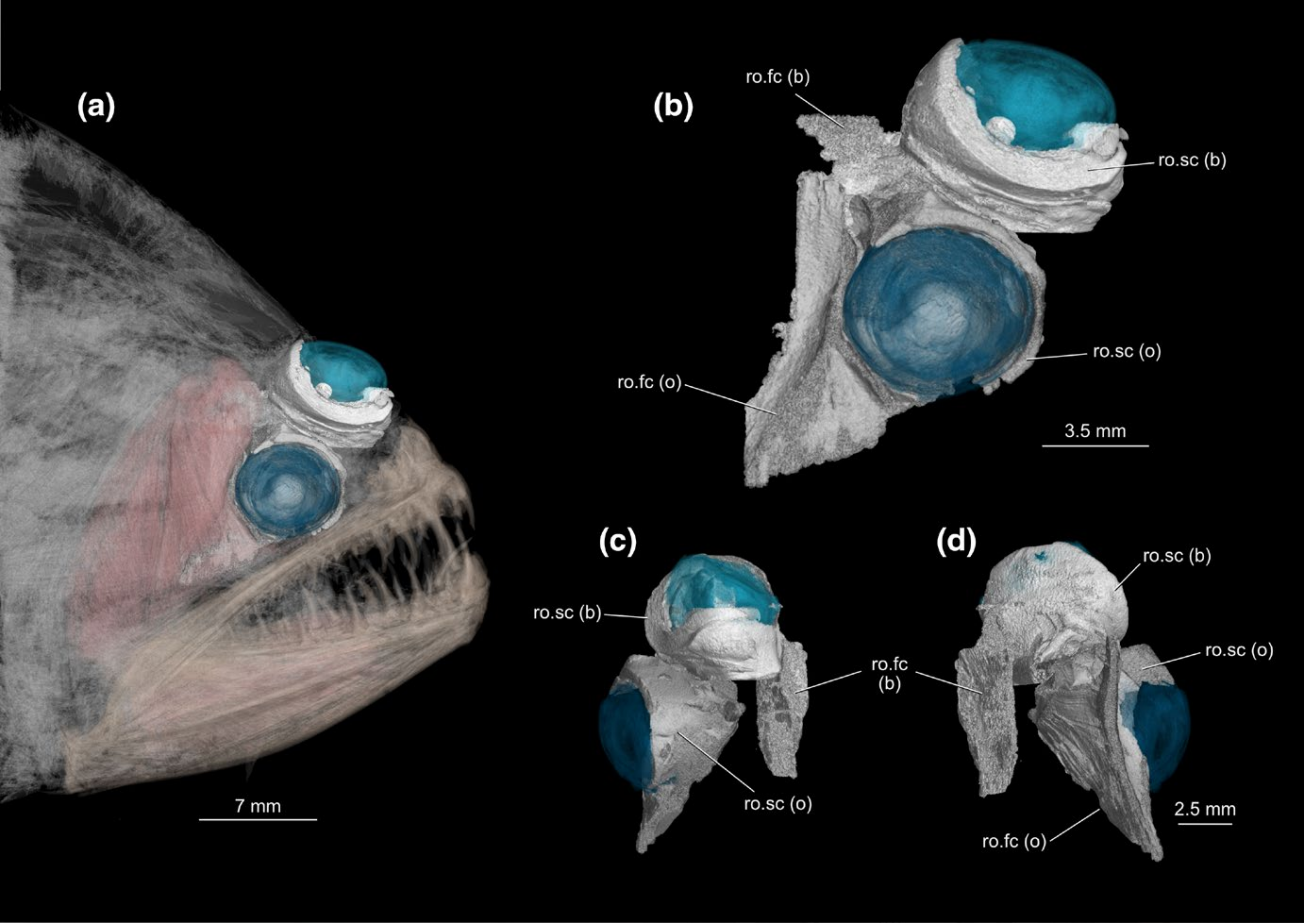
X‐ray microcomputed tomographies of the head of *Psettodes erumei* showing the position of the *recessus orbitalis* of both the ocular and blind sides. (a) Ocular side of the head, (b) ocular side of the eyes and their *recessus orbitales*, (c) frontal view, (d) posterior view. (b) blind side; (o) ocular side; ro.fc, *recessus orbitalis*, facial chamber; ro.sc, *recessus orbitalis*, scleral chamber.

The facial chamber associated with the migrated eye remains located on the blind side of the head in most flatfishes (Figures 1b, 6b and 7). The facial chamber of the blind side lies anterodorsal to the facial segment of the *adductor mandibulae* muscle and is usually larger than its counterpart on the ocular side (Figure 1b). Once the skin is removed, only the anterior portion of the blind side facial chamber is visible, with most of its body lying underneath the facial segment of the *adductor mandibulae*, sandwiched between this muscle and the hyopalatine arch (Figure 6b). The connection with the scleral chamber is more anterodorsal than on the ocular side, traversing the neurocranium through a space between the blind side frontal, parasphenoid, lateral ethmoid, and autosphenotic or pterosphenoid (when present).

**FIGURE 8:**
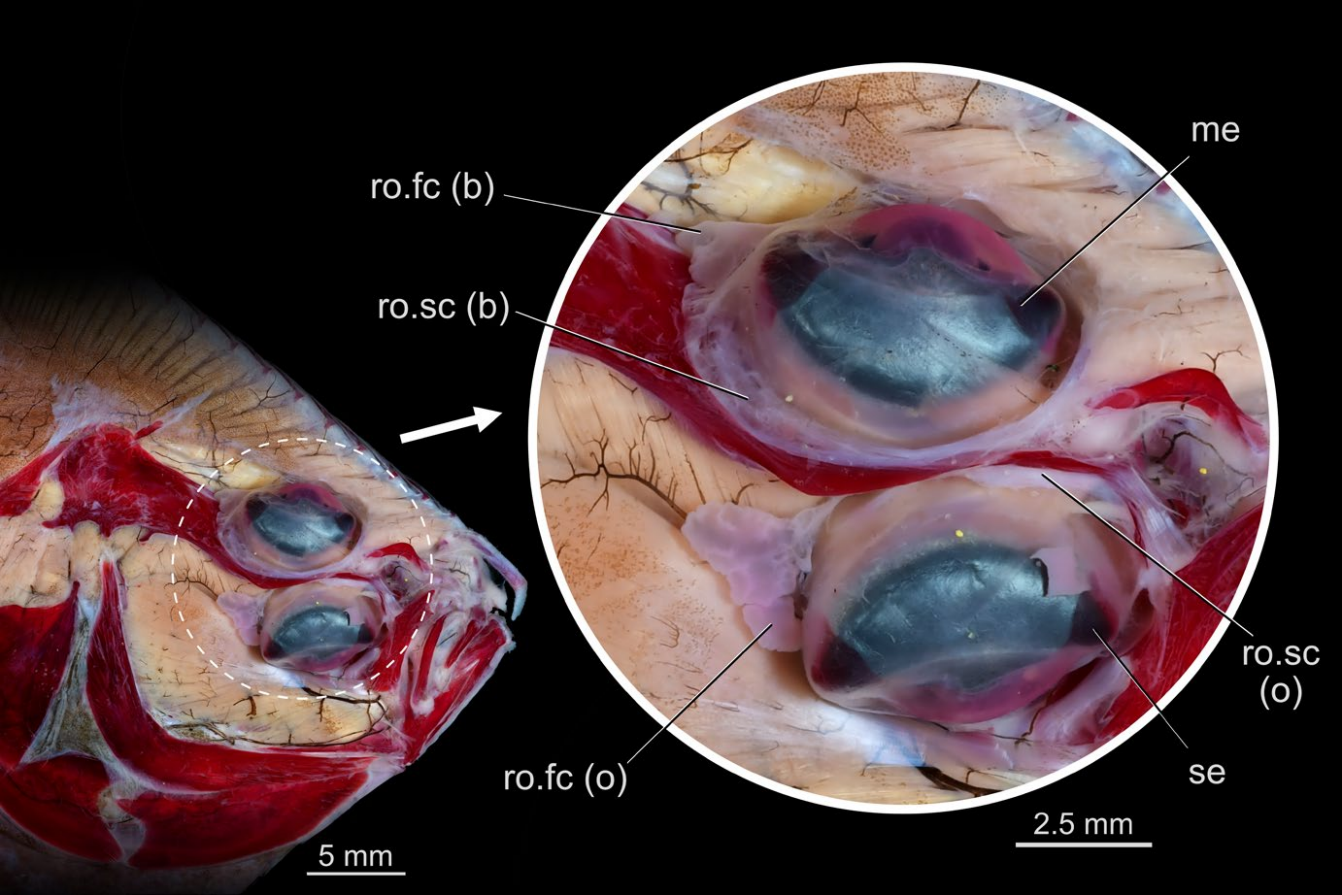
Ocular side of the head of *Rhombosolea tapirina* showing the position of the *recessus orbitalis* serving both the sessile and migrated eyes. (b) blind side; me, migrated eye; (o) ocular side; ro.fc, *rrecessus orbitalis*, facial chamber; ro.sc, *rrecessus orbitalis*, scleral chamber; se, ssessile eye.

*Pelotretis flavilatus* (Rhombosoleidae), *Limanda ferruginea*, *Pleuronectes platessa*, and *Pleuronichthys coenosus* (Pleuronectidae) are unique in having an extra section of the facial chamber anterior to both eyes. In *Rhombosolea tapirina* and *Peltorhamphus novaezeelandiae* (Rhombosoleidae), the blind side facial chamber is located on the ocular side of the head, immediately posterodorsal to the migrated eye (Figure 8). *Pegusa lascaris* (Soleidae) displays a unique condition in which the ocular side facial chamber is sectioned into dorsal and ventral subdivisions (Figure 1). The dorsal section is larger and surpasses the neurocranium dorsally. The ventral section is closer to the facial segment of the *adductor mandibulae* muscle and to the sessile eye. The blind side facial chamber of *P. lascaris* is externally undivided, as in the typical condition of other flatfishes. In *Hippoglossus hippoglossus*, the facial chamber of the blind side is partially subdivided into anterior and posterior sections.

**FIGURE 9:**
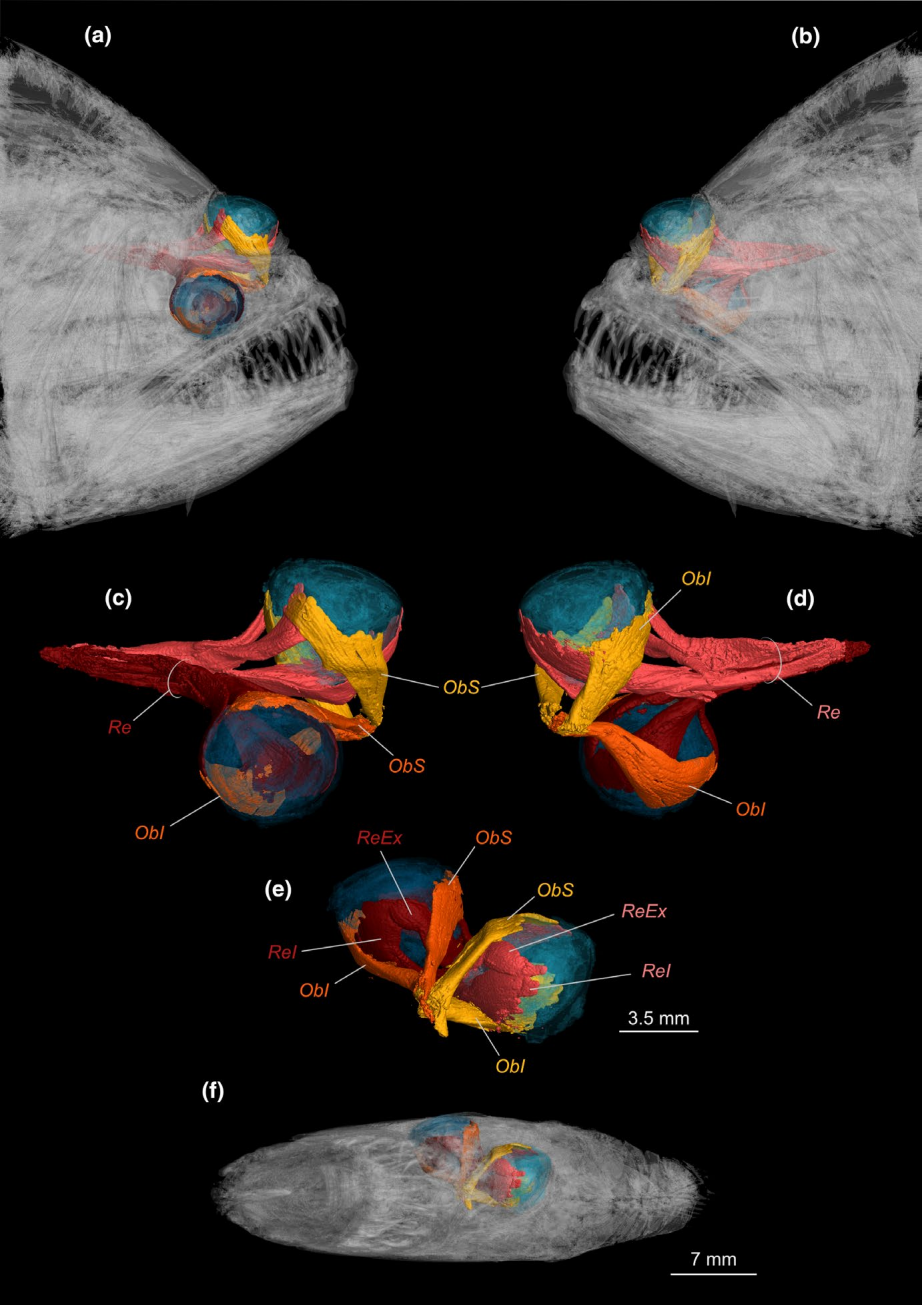
X‐ray microcomputed tomographies of the head of *Psettodes erumei* showing the position of the eyes and their associated musculature. (a) Ocular side of the head. (b) Blind side of the head. (c) Ocular side of the eyes and their corresponding muscles. (d) Blind side of the eyes and their corresponding muscles. (e) Frontal view of the eyes and their corresponding muscles. (f) Frontal view of the head. ObI, *obliquus inferioris*; ObS, *obliquus superioris*; Re, *recti* muscles; ReEx, *rectus externus*; ReI, *rectus inferioris*.

**FIGURE 10:**
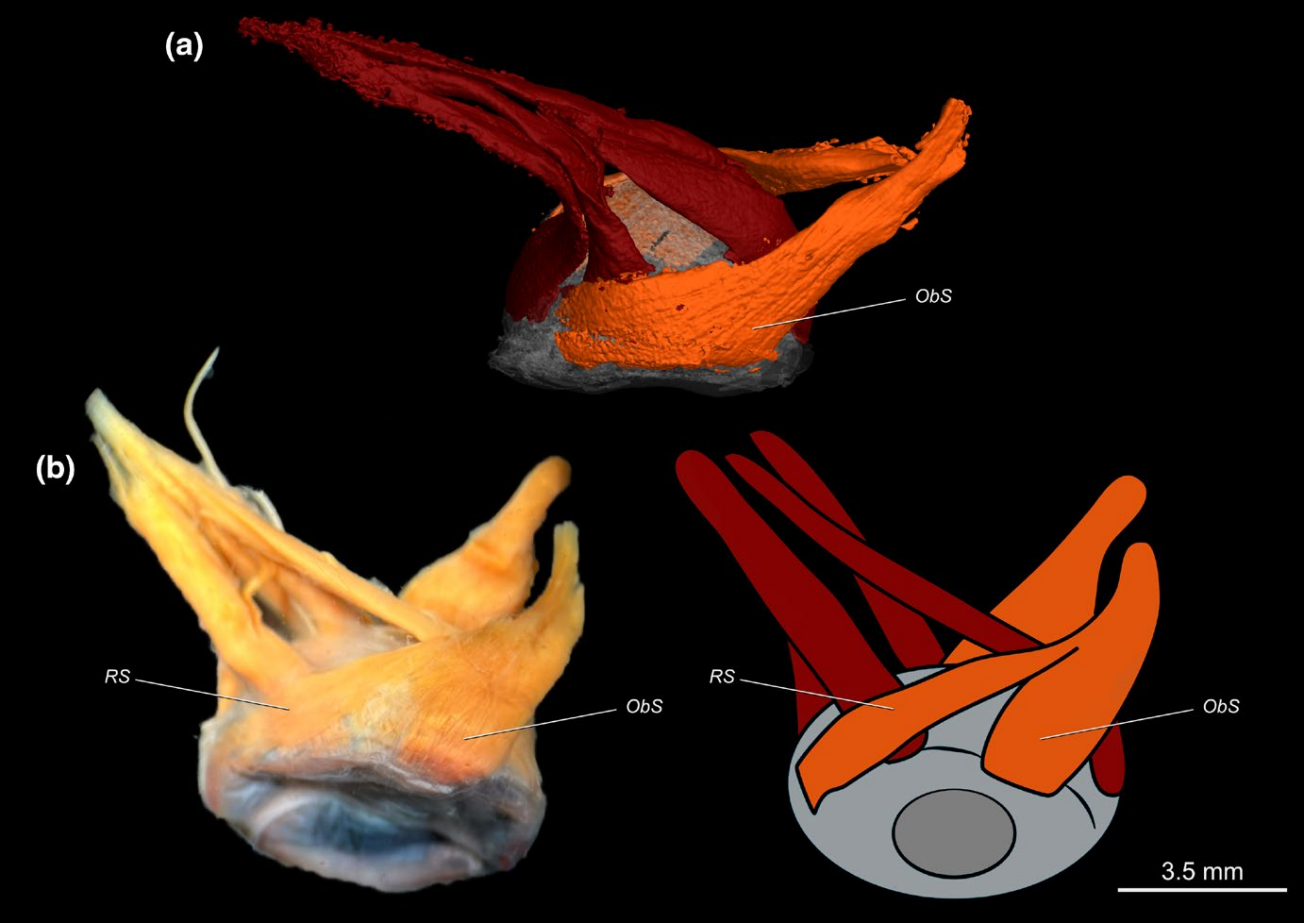
Dorsal view of the sessile (right) eye. (a) *Psettodes erumei*, X‐ray microcomputed tomography. (b) *Hippoglossus hippoglossus*, photography and corresponding schematic view. ObS, *obliquus superioris*; RS, rotatory slip.

In adult flatfishes, all extrinsic eye muscles are rearranged due to eye migration, which places the contralateral *obliqui* and *recti* muscles approximately perpendicular to each other (Figure 9). The *recti* muscles are considerably longer than the *obliqui*, having enough contraction length to move the protruded eye (Figure 9). Most pleuronectiforms have a specialization in the *obliquus superioris*. This specialization is visible near the insertion of the muscle, where it expands to form an additional, separate posterior section termed the rotatory slip by Cole and Johnstone (1902) (Figure 10). This section is absent in psettodids, achirids except *Gymnachirus nudus*, cynoglossids, paralichthodids, the rhombosoleid *Peltorhamphus novaezeelandiae*, and the soleids *Brachirus orientallis*, *Achiroides melanorhynchus*, *Aesopia cornuta*, *Aseraggodes dubius*, *Liachirus melanospilos*, and *Zebrias zebrinus*.

### 3.2 Functional inferences

**FIGURE 11:**
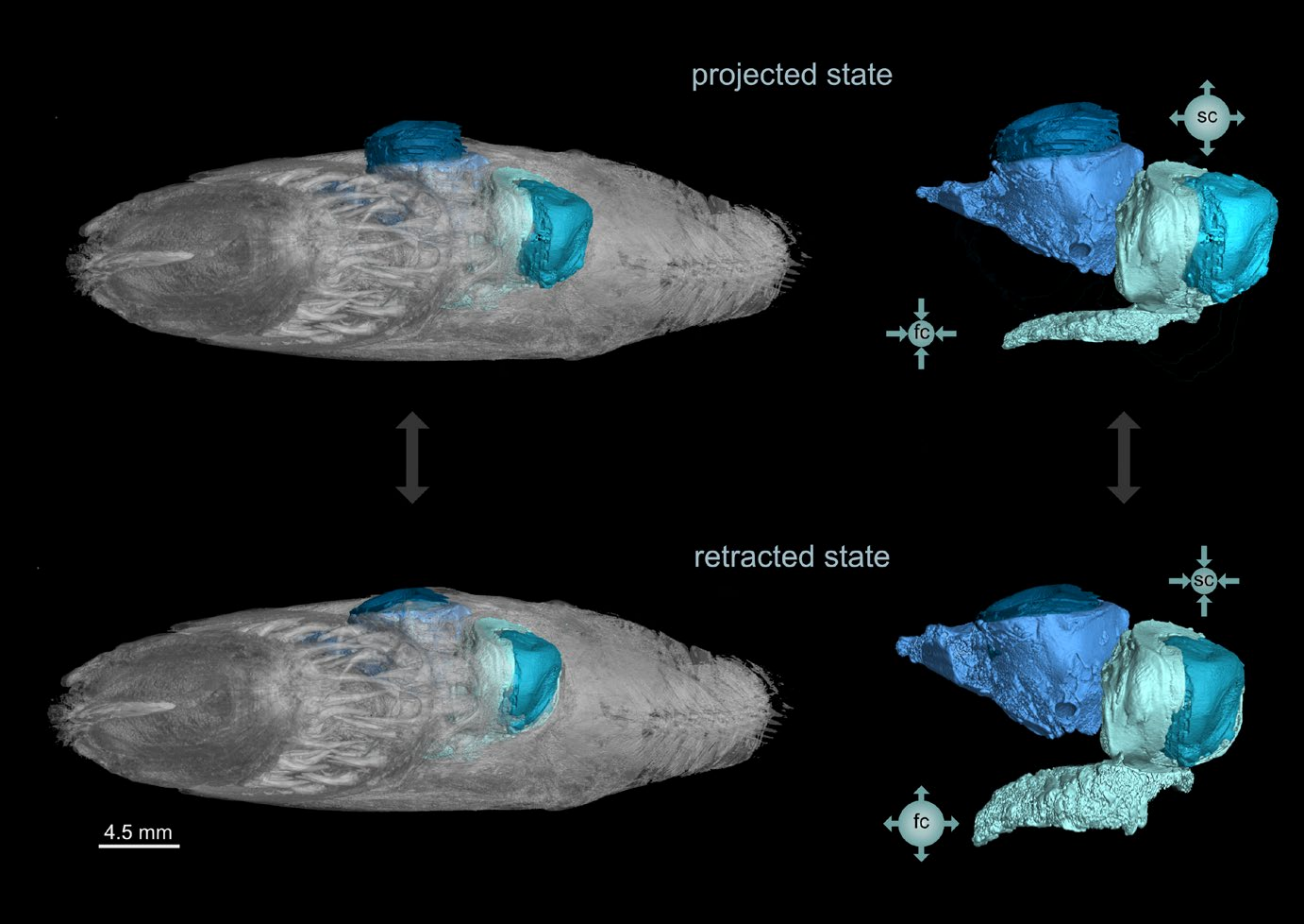
X‐ray microcomputed tomographies of the frontal view of the head of *Psettodes erumei*. Schematic representation of how the double‐pump mechanism of the two chambers of the *recessus orbitalis* work. fc, facial chamber; sc, Scleral chamber.

Morphological analysis combined with field observations allows us to infer the mechanisms of action of the *recessus orbitalis* in eye protrusion. In the retracted state, the smooth muscle fibers surrounding the scleral chamber of the *recessus orbitalis* and, possibly, all ocular muscles are contracted. This would move the interstitial fluid away from the scleral chamber, creating accommodation space for the eyeball. The fluid would then be concentrated in the facial chamber, which would have its smooth muscle fibers in a relaxed state (Figure 11). In some flatfishes, the expansion of the facial chamber can be observed externally. In response to an external stimulus approaching its eye, *Achirus lineatus* rapidly retracts the eyeball into the scleral chamber, a movement followed by a noticeable swelling of the region posterior to the eye (Movie S1). This area covers the ocular side facial chamber of the *recessus orbitalis*, indicating that this chamber expands due to the influx of interstitial fluid during eye retraction. During eye protrusion, the opposite actions take place: the smooth muscle fibers of the facial chamber contract and the fibers of the scleral chamber and ocular muscles relax (Figure 11). This makes the fluid flow from the facial to the scleral chamber, elevating the eye above head level. From an external view, the swollen area covering the ocular side facial chamber dissipates while the eye protrudes (Movie S1). The presence of a rippled elastic texture on the lateral outer margin of the orbit indicates that the eyes have enough skin to protrude dorsally above the body level, and when retracted, the skin loosens and is housed in the scleral chamber (Figure 3c).

## 4 DISCUSSION

### 4.1 Morphology and function

The *recessus orbitalis* was first described by Holt (1894), which remained until now the most detailed anatomical analysis of this unique structure. However, neither that nor any subsequent study realized that the organ comprised two chambers. Past studies identified only the herein termed facial chamber of the organ and considered that it opened through an orifice directly into the orbital cavity (Holt, 1894). Our analysis demonstrates that such an orifice is actually the interchamber constriction that connects the facial chamber to a second, completely closed chamber that is associated with the external surface of the sclera. This second chamber—the scleral chamber—is extremely delicate and difficult to visualize by direct dissection. This probably explains why it has gone unnoticed in all previous studies.

The muscular nature of the *recessus orbitalis* has also never been detailed. According to Holt (1894), the facial chamber was marked by “white muscular bands” attaching to its inner surface. Cole and Johnstone (1902) state that it was possible to see “bands of muscle fibers which cross it and are massed together on the internal wall.” Our histological sections show that these muscles are smooth, not skeletal, and that they were not confined to the facial chamber but also extended into the scleral chamber (Figures 2, 3, 4, 5). Smooth muscle fibers operate under the contraction–relaxation balance via phosphorylation of the myosin light chain kinase (MLCK) mediated by the presence/absence of Ca^2+^ (Kilarski, 2019; Webb, 2003). The muscle will remain contracted as long as Ca^2+^ is bound to calmodulin and the MLCK is phosphorylated (Webb, 2003). As a result, smooth muscles are capable of maintaining contraction for prolonged periods with little energy expenditure (Butler & Siegman, 2010; Webb, 2003). This property is consistent with the inferred double‐pump mechanism of action of the *recessus orbitalis*, which requires prolonged contraction of the fibers of some of its chambers to keep the eye either fully protruded or retracted. Similar mechanisms of prolonged smooth muscle contraction are found in blood vessels (Webb, 2003) and in the adductor muscle that bivalves use to keep their shells closed (Butler & Siegman, 2010).

### 4.2 Distribution and evolutionary implications

This is the first time in which all 16 families of flatfishes have been thoroughly dissected in order to document the morphology of the *recessus orbitalis*. Campbell et al. (2020) documented the distribution of the *recessus orbitalis* in pleuronectiforms, but the authors analyzed only 18 species from 10 families by superficial dissections of fresh specimens, which usually makes it more difficult to visualize translucent structures such as the *recessus orbitalis*. For the remaining six families, the authors assumed the presence of the *recessus orbitalis* by analyzing photographs of only three of those families (Achiridae, Cyclopsettidae, and Rhombosoleidae) in which the eyes seemed to be protruded. Achiropsettidae, Oncopteridae, and Paralichthodidae were not analyzed either by photographs or direct dissection. The authors failed to find the *recessus orbitalis* in the Psettodidae they dissected, and trusted the statement of Chabanaud (1937) that *Psettodes* is incapable of protruding its eyes. They consequently concluded that the presence of the *recessus orbitalis* was synapomorphic for Pleuronectoidei. Chapleau (1993), on the other hand, although not examining the structure in *Psettodes*, assumed that the *recessus orbitalis* was universal to all flatfishes and proposed that the structure was a synapomorphy for Pleuronectiformes.

In our survey, we confirmed via dissections, tomography, and histology that the *recessus orbitalis* is unequivocally present in all flatfish families, including Achiridae, Achiropsettidae, Cyclopsettidae, Oncopteridae, Paralichthodidae, Psettodidae, and Rhombosoleidae. Despite the drastic dorsal‐fin ray modification present on the blind side of *Oncopterus darwinii* (Presti et al., 2024), the *recessus orbitalis* of the blind side is normally placed as in other flatfishes. In *Psettodes*, the *recessus orbitalis* is not visible from a superficial dissection. Simply removing the skin is not enough to see the structure in this taxon since the *recessus orbitalis* is located beneath the facial segment of the *adductor mandibulae* (Figures 6a and 7), being sandwiched in between this muscle and the *adductor hyomandibulae* (Figure 6b). Underwater footage of living psettodids also shows that their eyes are considerably protruded above the surface of the head (Safaris, 2017). However, the *recessus orbitalis* of *Psettodes* seems relatively reduced compared to other flatfishes. Consequently, it probably stores less fluid, resulting in a moderate projection of the eyes beyond the head's level. Our discovery that the *recessus orbitalis* is indeed present in psettodids, as well as in all other flatfish families, confirms previous surmisings that the possession of this structure is a synapomorphy for Pleuronectiformes (Chapleau, 1993; Holt, 1894).

Holt (1894) argued that the eye is an organ of considerable weight and that the simple relaxation of the ocular muscles would not be enough to project it. Therefore, the evolution of the *recessus orbitalis* probably had profound implications for flatfishes. The protrusion of the eye in the group is thought to help vision due to the benthic lifestyle of flatfishes, maintaining sight while buried or camouflaged in the substrate (Campbell et al., 2020; Cole & Johnstone, 1902; Holt, 1894; Norman, 1934). By elevating their eyes, their visual field is enhanced, facilitating the search for resources or preventing being caught by predators. Although the *recessus orbitalis* is unique to flatfishes, eye elevation is a common adaptation shared with other benthic fishes such as mudskippers (Aiello et al., 2023; Stebbins & Kalk, 1961), Uranoscopids, *Lophius* (pers. obs.), and even octopuses (Hanke & Kelber, 2020). Mudskippers (Gobiiformes, Oxudercidae) are amphibious fishes that live in mangroves and tidal flats of the western Indo‐Pacific and eastern Atlantic oceans, and these fishes naturally have their eyes projected and blink (i.e., retract their eyes ventrally) when necessary (Aiello et al., 2023). In mudskippers, the eye is retracted by the joint action of all six extrinsic ocular muscles, lowering it until reaching a dermal cup, which in turn passively moves dorsally to meet the dorsal margin of the eye. Once the muscles relax, the elastic nature of the dermal cup helps the eye to rise again (Aiello et al., 2023).

The blinking behavior of mudskippers is associated with wetting, cleaning, and protecting the eye, functions convergent with the eyes of tetrapods (Aiello et al., 2023; Ogimoto et al., 2021). Aiello et al. (2023) further hypothesized that blinking behavior might have originated in aquatic environments and secondarily exapted for terrestrial environments. In fishes, the ability to retract eyes into sockets has evolved at least four times; these include whale shark (*Rhincodon typus*), the giant guitarfish (*Rhynchobatus djiddensis*), the fine‐patterned pufferfish (*Takifugu flavipterus*), and the mudskippers (Oxudercidae) (Aiello et al., 2023; Ogimoto et al., 2021; Tomita et al., 2016; Tomita et al., 2020). Although flatfishes do not completely occlude their eyes like the examples above, the ability to retract the eyes is to some extent comparable to such mechanisms (Club, 2009). Flatfishes retract their eyes for protection in response to a threat (Holt, 1894) and probably to clean the eyes from sand particles.

In contrast to mudskippers, most Pleuronectiformes have an extra section of the *obliquus superioris*: the rotatory slip (Figure 10), which was first reported by Cole and Johnstone (1902). This muscle section is absent in Psettodidae, Achiridae (except *Gymnachirus nudus*), Cynoglossidae, Paralichthodidae, *Peltorhamphus novaezeelandiae* (Rhombosoleidae), and some Soleidae (*Brachirus orientallis*, *Achiroides melanorhynchus*, *Aesopia cornuta*, *Aseraggodes dubius*, *Liachirus melanospilos*, and *Zebrias zebrinus*). However, the insertion of the *obliquus superioris* on the eyeball is still wider in these taxa when compared to nonflatfishes. The rotatory slip and extended insertion of the muscle probably provide additional rotational range to the eye, increasing the ability to explore wide angles of vision when the eyes are protruded.

## AUTHOR CONTRIBUTIONS

PP: Conceptualization; data curation; formal analysis; funding acquisition; investigation; methodology; project administration; validation; visualization; writing—original draft; writing—review and editing. GDJ: Formal analysis; resources; supervision; validation; writing—review and editing. AD: Conceptualization; data curation; formal analysis; funding acquisition; investigation; methodology; project administration; resources; supervision; validation; writing—original draft; writing—review and editing.

## CONFLICT OF INTEREST STATEMENT

The authors declare no competing interests.

## Supporting information


Movie S1.

## Data Availability

All data generated or analyzed during this study are included in this published article.
